# The Prognostic Value of Geriatric Nutritional Risk Index in Evaluating Rehospitalization and One-Year Mortality in Patients With Heart Failure

**DOI:** 10.7759/cureus.44460

**Published:** 2023-08-31

**Authors:** Ahmet Balun, Alkame Akgümüş, Zehra G Çetin, Bekir Demirtaş

**Affiliations:** 1 Cardiology, Bandırma Onyedi Eylül University, Balıkesir, TUR; 2 Cardiology, Ankara Bilkent City Hospital, Ankara, TUR; 3 Cardiology, Ankara Etlik City Hospital, Ankara, TUR

**Keywords:** mortality, nutrition, reduced ejection fraction, rehospitalization, geriatric nutritional risk index, malnutrition, heart failure

## Abstract

Background: Malnutrition is frequently observed in patients with heart failure, and malnutrition causes poor prognosis in these patients. Various calculation tools are used to assess malnutrition, with the geriatric nutritional risk index (GNRI) being one that is frequently used. In our study, we aimed to investigate the value of GNRI in assessing one-year mortality and rehospitalization in patients with heart failure.

Method:A total of 196 patients aged 60 years and older were included in this retrospective study. A GNRI ≤ 98 was defined as malnutrition. Patients were divided into two groups: GNRI ≤ 98 (malnutrition) and GNRI > 98 (non-malnutrition). Rehospitalization due to heart failure and mortality were compared between both groups in the one-year follow-up.

Results: The duration of hospitalization was significantly lower in the malnourished group compared to the non-malnutrition group (11.5 ± 7.5 days vs. 20.9 ± 16.3 days). All-cause mortality was significantly higher in the malnutrition group (30.8% vs. 18.1, p = 0.045). Risk factors were evaluated to predict all-cause death by Cox regression analysis, and GNRI (hazard ratio (HR) = 0.968; 95%CI: 0.942-0.995; p = 0.018) was associated with all-cause mortality.

Conclusions: GNRI, which is used as an indicator of malnutrition, is associated with all-cause mortality at one-year follow-up. Higher mortality was observed in the group with low GNRI, but it was observed that this group was hospitalized for less time due to heart failure.

## Introduction

The incidence of heart failure increases as the population ages, and it has become a major public health concern worldwide due to its high mortality and morbidity rates. Despite advancements in medical treatments, the health system continues to be strained by recurrent hospitalizations caused by heart failure. Previous research indicates that around 60% of heart failure patients pass away within five years of their initial diagnosis [1]. It was observed that about 26% of the patients hospitalized for heart failure were hospitalized again within one month after discharge [2].

As in many diseases, malnutrition has great prognostic importance in heart failure patients. Previous studies have shown that patients with poor nutritional status have a high one-year all-cause mortality rate [3,4]. Since heart failure is seen in the elderly population, it causes malnutrition to be seen more frequently in these patients. At the same time, nutritional status is affected due to intestinal edema and anorexia. The nutritional status of heart failure patients is evaluated using various nutritional indices and body mass index (BMI). Geriatric nutrition risk index (GNRI) is calculated by considering both the BMI and serum albumin level of the patient, and it has been shown that it can predict the prognosis in patients with heart failure [5,6]. When calculating GNRI, adding the serum albumin level to the BMI makes it a more precise nutritional indicator. This is because it reduces the likelihood of errors resulting from weight gain that could cause miscalculations due to fluid overload in patients. Specifically, it helps to counteract the increase in BMI and decrease in albumin that can occur. In our study, we aimed to investigate the effect of GNRI on one-year mortality and rehospitalization in heart failure patients. In addition to similar studies in the literature, it is aimed to examine the effect of nutritional status on hospitalization times.

## Materials and methods

Trial design and population

**Figure 1:**
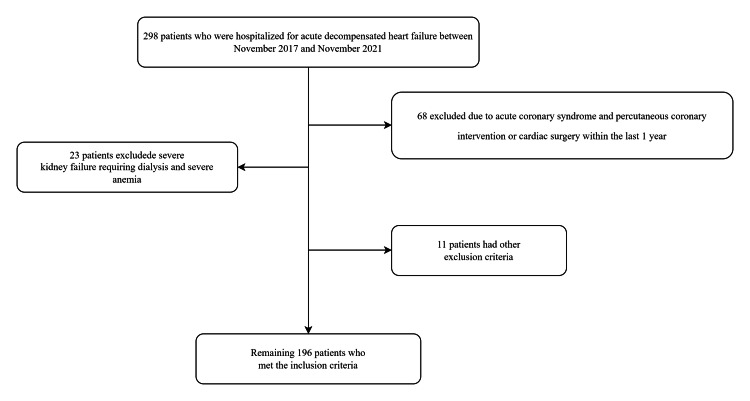
A flow-chart showing the number of included and excluded patients

A total of 196 patients aged 60 years and older were included in this retrospective study. Patients included those who were hospitalized for acute decompensated heart failure between November 2017 and November 2021 and followed up after discharge. The study was conducted in Bandırma Onyedi Eylül University in Balıkesir, Türkiye, and Ankara Bilkent City Hospital and Ankara Etlik City Hospital in Ankara, Türkiye. The Ethics Committee of Ankara City Hospital approved the study (approval number: E2-23-4301), and the study was conducted in accordance with the Declaration of Helsinki. The detailed exclusion criteria were congenital heart disease, severe kidney failure requiring dialysis, patients on parenteral nutrition, severe liver function disorder, severe anemia, hyperthyroidism and hypothyroidism, presence of active infection, chronic inflammatory disease, acute coronary syndrome within the last one year, and percutaneous coronary intervention or cardiac surgery within the last one year. Figure 1 shows the process of selection of study participants. All patients were being treated with the maximum tolerable dose of beta-blockers, angiotensin-converting enzyme inhibitors/angiotensin II receptor blockers, mineralocorticoid receptor antagonists, and other diuretics.

The clinical laboratory and demographic data of the patients were obtained from the hospital registry system. The patients were followed up for one year afterward. All-cause mortality and hospitalizations due to heart failure were obtained from the national health data registry system. The number of hospitalizations due to heart failure, the number of recurrent hospitalizations, and the total hospital stay in these hospitalizations were examined in the one-year follow-up after the patients were discharged without symptoms except for those patients who could not be followed up for one year due to death.

GNRI

The data needed to calculate GNRI for the patients was taken from their file at the first hospitalization, and the height, weight, and BMI were calculated. Then, GNRI was calculated with the formula 14.89 × albumin (g/dL) + 41.7 × BMI/22. In previous studies, a GNRI ≤ 98 was defined as malnutrition [7]. In our study, patients were divided into two groups: GNRI ≤ 98 (malnutrition) and GNRI > 98 (non-malnutrition).

Statistical analysis

IBM SPSS Statistics for Windows, Version 23.0 (IBM Corp., Armonk, New York, United Sta) was used for statistical analysis. All variables were tested for normal distribution. Continuous variables were expressed as mean ± standard deviation and categorical variables were expressed as numbers (percentage). Normally distributed continuous variables were compared using Student’s t-test for means. Categorical variables are shown as numbers and percentages and were compared using the chi-square test and Fisher’s exact test. Cox proportional hazard models were used to evaluate, after adjusting for prespecified clinically relevant comorbidity and demographic variables. All tests were two-sided, and p values < 0.05 were considered significant.

## Results

There were 196 patients in the study group. The patients were divided into two groups: (i) 52 patients with malnutrition with a GNRI ≤ 98 and (ii) 144 patients without malnutrition, that is, with a GNRI > 98. The mean age of all patients was 72.9 ± 11.1 years and 53.1% were male. BMI was statistically significantly lower in the malnourished group than in the non-malnutrition group (22.2 ± 1.9 kg/m^2^ vs. 29.6 ± 4.9 kg/m^2^, p < 0.001). Serum albumin level was statistically lower in the group with malnutrition than that in the group without malnutrition (3.4 ± 0.3 g/dL vs. 3.9 ± 0.4 g/dL, p < 0.001). Serum sodium level was significantly lower in the malnourished group (135.5 ± 3.6 mmol/L vs. 138.7 ± 3.5 mmol/L, p < 0.001). Other clinical laboratory and demographic characteristics are shown in Table 1.

**Table 1:** Demographic, clinical, and laboratory characteristics of the patients GNRI: Geriatric Nutritional Risk Index; BMI: Body Mass Index; LvEF: Left Ventricular Ejection Fraction; SGLT-2, Sodium-glucose cotransporter protein-2 inhibitors

Variables	All (n=196)	GNRI ≤ 98 (n=52)	GNRI > 98 (n=144)	p-value
Age (years), mean±SD	72.9 ± 11.1	76.0 ± 11.6	71.8 ± 10.7	0.027
Sex (Male), n (%)	104 (53.1)	32 (61.5)	72 (50)	0.195
Hypertension, n (%)	105 (53.6)	30 (57.7)	75 (52.1)	0.520
Diabetes mellitus, n (%)	61 (31.1)	12 (23.1)	49 (34.0)	0.165
Ischemic etiology, n (%)	145 (74)	36 (69.2)	109 (75.7)	0.232
BMI (kg/m^2^), mean±SD	27.6 ± 5.4	22.2 ± 1.9	29.6 ± 4.9	<0.001
LvEF (%), mean±SD	33.8 ± 5.1	33.9 ± 4.1	33.8 ± 5.4	0.811
SGLT-2, n (%)	48 (24.5)	37 (25.7)	11 (21.2)	0.514
Hemoglobin (g/dL), mean±SD	13.0 ± 1.6	12.9 ± 1.6	13.0 ± 1.6	0.701
Lymphocyte count (x1000/uL), mean±SD	11.5 ± 3.6	10.8 ± 3.6	11.8 ± 3.6	0.063
Platelet count (x1000/uL), mean±SD	235.2 ± 66.9	234 ± 68.4	235.6 ± 66.7	0.882
Creatinine (mg/dL), mean±SD	1.2 ± 0.2	1.2 ± 0.2	1.2 ± 0.2	0.172
Serum albumin (g/dL), mean±SD	3.8 ± 0.4	3.4 ± 0.3	3.9 ± 0.4	<0.001
Potassium (mmol/L), mean±SD	4.0 ± 0.3	4.1 ± 0.3	4.0 ± 0.3	0.432
Sodium (mmol/L), mean±SD	137.9 ± 3.8	135.5 ± 3.6	138.7 ± 3.5	<0.001
GNRI, mean±SD	109.0 ± 12.5	93.1 ± 3.6	114.6 ± 9.2	<0.001

In Table 2, recurrent hospitalizations, lengths of hospital stay, and all-cause mortality in the one-year follow-up of the patient groups are presented. Considering the hospitalizations of all patients, it was observed that 52% were not hospitalized again for one year, but 32.7% were hospitalized for heart failure more than once. The duration of hospitalization was significantly lower in the malnourished group compared to the non-malnutrition group (11.5 ± 7.5 days vs. 20.9 ± 16.3 days). All-cause mortality was significantly higher in the malnutrition group (30.8% vs. 18.1, p = 0.045).

**Table 2:** Clinical outcomes of the groups at the one-year follow-up GNRI: Geriatric Nutritional Risk Index

Variables	All (n=196)	GNRI ≤ 98 (n=52)	GNRI > 98 (n=144)	p-value
Rehospitalization				
None, n (%)	102 (52.0)	32 (61.5)	70 (48.6)	0.200
1 time, n (%)	30 (15.3)	8 (15.4)	22 (15.3)	
>1 times, n (%)	64 (32.7)	12 (23.1)	52 (36.1)	
Length of stay (days), mean±SD	18.9 ± 15.3	11.5 ± 7.5	20.9 ± 16.3	0.014
All-cause mortality	42 (21.4)	16 (30.8)	26 (18.1)	0.045

Table 3 shows the risk factors that were evaluated to predict all-cause death by Cox regression analysis. Age (hazard ratio (HR) = 1.039; 95%CI: 1.008-1.072; p = 0.014), serum potassium level (HR = 3.301; 95%CI: 1.307-8.337; p = 0.012), and GNRI (HR = 0.968; 95%CI: 0.942-0.995; p = 0.018) were associated with all-cause mortality.

**Table 3:** Cox regression analysis for the risk factors in predicting the all-cause mortality HR: Hazard Ratio; CI: Confidence Interval; LvEF: Left ventricular ejection fraction; GNRI: Geriatric Nutritional Risk Index

	Univariate, HR (95%CI)	p-value	Multivariate, HR (95%CI)	p-value
Age, Year	1.048 (1.017-1.080)	0.002	1.039 (1.008-1.072)	0.014
LvEF	1.067 (1.000-1.067)	0.051	1.064 (0.994-1.138)	0.073
Potassium	3.215 (1.238-8.348)	0.016	3.301 (1.307-8.337)	0.012
Sodium	0.935 (0.866-1.008)	0.080	0.971 (0.894-1.056)	0.493
GNRI	0.960 (0.935-0.985)	0.002	0.968 (0.942-0.995)	0.018
Ischemic etiology	0.431 (0.182-1.024)	0.057	0.456 (0.191-1.089)	0.077

## Discussion

To the best of our knowledge, this is the first study in the literature to examine the relationship between nutritional status and mortality in heart failure patients as well as hospitalizations. The nutritional status was evaluated in our study with GNRI, and it was observed that all-cause mortality was lower in patients with better nutritional status (high GNRI value). After conducting evaluations of patients with and without malnutrition, it was discovered that there was no significant difference in the number of hospitalizations during their one-year follow-up. However, it was observed that the group without malnutrition had a longer total length of hospital stay.

The GNRI was first used as an indicator of malnutrition to show the in-hospital mortality of elderly patients [8]. In addition, studies have also shown its prognostic value in many disease groups such as chronic kidney disease and cancer [9,10]. In cardiac diseases, its prognostic value has been shown in patients with coronary artery disease, transcatheter aortic valve implantation, and heart failure [11,12]. In these studies, the GNRI was associated with long-term mortality in elderly patients with acute coronary syndrome and in heart failure patients, while it was associated with prognosis and mortality in patients with transcatheter aortic valve implantation.

Malnutrition is frequently observed in heart failure patients due to changes in metabolism and gastrointestinal dysfunction. The low level of albumin caused by malnutrition affects the osmotic pressure in patients with heart failure, causing edema in the gastrointestinal tract and malabsorption, both of which increase malnutrition and reduce the absorption of diuretics. BMI is one of the most common indicators of malnutrition and defines which people are obese. Although obesity is an independent risk factor for the development of heart failure, the obesity paradox also shows greater survival in heart failure [13]. The paradox of obesity in heart failure is a valid survival paradox and has been attributed to a variety of complex causes [14-16]. Considering BMI is only an anthropometric measurement, it is not an accurate indicator of nutritional status, and it has been shown to be prognostic in heart failure patients with the definition of obesity paradox. Albumin is used in addition to BMI in calculating GNRI. Albumin is a serum protein, which alone can be used as an indicator of nutritional status. However, in decompensated heart failure, serum albumin levels may decrease due to increased extracellular fluid volume [17]. Using BMI and serum albumin together, however, while calculating GNRI minimizes the effect of confounding factors such as increased fluid volume. For these reasons, it was thought that GNRI showed better nutritional status than BMI, and its prognostic importance was shown in heart failure patients. Similar to other studies conducted with GNRI, a higher one-year mortality was observed in patients with malnutrition in our study, and GNRI was shown to be an independent predictor of mortality [3,4,11].

There is not enough data in the literature on the effect of GNRI on hospital readmission and length of stay in heart failure patients. In a study by Aziz et al., patients with heart failure were evaluated with the Nutritional Risk Index and it was shown that those with low nutritional risk index scores had longer hospital stays and higher rates of readmission with episodes of acute decompensated heart failure [18]. The Nutritional Risk Index in their study used albumin, similar to the calculation tool used in our study, but unlike BMI, ideal body weight was used. However, the malnourished group in our study was older and had a lower BMI. In the current study, no significant difference was found between the groups with regard to the rates of readmission to the hospital due to heart failure after discharge. However, when the one-year hospitalization period due to heart failure was evaluated, it was seen that the group with malnutrition stayed in the hospital for a shorter period. This difference in our study was attributed to the higher BMI of patients without malnutrition, and therefore to the higher obesity rate of patients without malnutrition.. Given that obesity leads to an earlier onset of symptoms of heart failure, it is thought that it may lead to a later regression of symptoms in the treatment phase [19]. In previous studies, the concept of obesity paradox was expressed, and the deterioration of the quality of life in obese individuals and the fact that heart failure symptoms affect these individuals more functionally may cause this [13].

Hypokalemia and hyperkalemia in heart failure are common as a result of drugs used to treat renin-angiotensin-aldosterone system dysfunction and poor kidney function. Increasing and decreasing potassium levels during follow-up in heart failure patients are important because they change medical treatment preferences. It has also been shown in previous studies that dyscalemia has prognostic importance in heart failure [20,21]. However, arrhythmias caused by dyscalemia seriously affect the prognosis in heart failure patients. In our study, it was shown that serum potassium level is associated with all-cause mortality in heart failure patients. The reason why the increase in potassium level was found to be associated with mortality in our study was thought to be due to the fact that the GNRI calculation and laboratory values were calculated with venous blood samples taken at the first hospitalization of these patients, and that patients with hyperkalemia had reservations about using mineralocorticoid receptor antagonists.

There are several limitations in this study. First, this study was a retrospective study. Second, the changes in BMI and serum albumin levels used in the calculation of the nutritional index during follow-up were not taken into account, akin to similar studies in the literature. Finally, studies with large populations and different nutritional status indicators are needed to investigate the effect of nutritional status in heart failure patients, and the effect of these nutritional status scores on scores assessing comorbidity status should be evaluated as well [22].

## Conclusions

Malnutrition is common in patients with heart failure. One of the tools that shows the nutritional status of heart failure patients is GNRI. In our study, it was shown that GNRI is a predictor of one-year all-cause mortality in heart failure patients. When the number of hospitalizations for one year in the two groups of heart failure patients was evaluated, no significant difference was found between the malnourished group and the non-malnutrition group. However, it was observed that the group without nutritional deficiencies stayed in the hospital for a longer period of time due to heart failure within one year.

## References

[1] Bottle A, Newson R, Faitna P, Hayhoe B, Cowie MR (2023). Risk prediction of mortality for patients with heart failure in England: observational study in primary care. ESC Heart Fail.

[2] Namvar M, Fakhrolmobasheri M, Mazaheri-Tehrani S, Heidarpour M, Emamimeybodi M, Shafie D (2023). Association between the length of hospital stay and 30-day outcomes in patients admitted with acute decompensated heart failure. Emerg Med Int.

[3] Yoshihisa A, Kanno Y, Watanabe S (2018). Impact of nutritional indices on mortality in patients with heart failure. Open Heart.

[4] Cheng YL, Sung SH, Cheng HM, Hsu PF, Guo CY, Yu WC, Chen CH (2017). Prognostic nutritional index and the risk of mortality in patients with acute heart failure. J Am Heart Assoc.

[5] Liu L, Chen Y, Xie J (2022). Association of GNRI, NLR, and FT3 with the clinical prognosis of older patients with heart failure. Int Heart J.

[6] Sze S, Pellicori P, Zhang J, Clark AL (2019). Malnutrition, congestion and mortality in ambulatory patients with heart failure. Heart.

[7] Minamisawa M, Seidelmann SB, Claggett B (2019). Impact of malnutrition using geriatric nutritional risk index in heart failure with preserved ejection fraction. JACC Heart Fail.

[8] Bouillanne O, Morineau G, Dupont C (2005). Geriatric nutritional risk index: a new index for evaluating at-risk elderly medical patients. Am J Clin Nutr.

[9] Cho A, Park SY, Cha YS, Park HC, Kim DH, Lee YK (2022). The change in geriatric nutritional risk index is associated with mortality in patients who start hemodialysis: Korean Renal Data Registry, 2016-2018. Sci Rep.

[10] Miao X, Ding L, Hu J (2023). A web-based calculator combining geriatric nutritional risk index (GNRI) and Tilburg frailty indicator (TFI) predicts postoperative complications among young elderly patients with gastric cancer. Geriatr Gerontol Int.

[11] Li Y, Shen J, Hou X (2023). Geriatric nutritional risk index predicts all-cause mortality in the oldest-old patients with acute coronary syndrome: a 10-year cohort study. Front Nutr.

[12] Koseki K, Yoon SH, Kaewkes D (2021). Impact of the geriatric nutritional risk index in patients undergoing transcatheter aortic valve implantation. Am J Cardiol.

[13] Horwich TB, Fonarow GC, Clark AL (2018). Obesity and the obesity paradox in heart failure. Prog Cardiovasc Dis.

[14] Oga EA, Eseyin OR (2016). The obesity paradox and heart failure: a systematic review of a decade of evidence. J Obes.

[15] Anker SD, Negassa A, Coats AJS, Afzal R, Poole-Wilson PA, Cohn JN, Yusuf S (2003). Prognostic importance of weight loss in chronic heart failure and the effect of treatment with angiotensin-converting-enzyme inhibitors: an observational study. Lancet.

[16] Kenchaiah S, Evans JC, Levy D (2002). Obesity and the risk of heart failure. N Engl J Med.

[17] Jones CH, Smye SW, Newstead CG, Will EJ, Davison AM (1998). Extracellular fluid volume determined by bioelectric impedance and serum albumin in CAPD patients. Nephrol Dial Transplant.

[18] Aziz EF, Javed F, Pratap B (2011). Malnutrition as assessed by nutritional risk index is associated with worse outcome in patients admitted with acute decompensated heart failure: an ACAP-HF data analysis. Heart Int.

[19] Lavie CJ, Alpert MA, Arena R, Mehra MR, Milani RV, Ventura HO (2013). Impact of obesity and the obesity paradox on prevalence and prognosis in heart failure. JACC Heart Fail.

[20] Ferreira JP, Butler J, Rossignol P (2020). Abnormalities of potassium in heart failure: JACC state-of-the-art review. J Am Coll Cardiol.

[21] Aldahl M, Jensen AC, Davidsen L (2017). Associations of serum potassium levels with mortality in chronic heart failure patients. Eur Heart J.

[22] Sonaglioni A, Lonati C, Rigamonti E (2022). CHA(2)DS(2)-VASc score stratifies mortality risk in heart failure patients aged 75 years and older with and without atrial fibrillation. Aging Clin Exp Res.

